# Mechanistic insights into promoted biogas production and reduced antibiotic resistance genes’ risks of dry anaerobic digestion of organic wastes with biochar addition

**DOI:** 10.1186/s40643-025-01003-2

**Published:** 2026-01-27

**Authors:** Zhenqi Wang, Min Zhang, Xiaoyong Qian, Yuanzhi Ni, Xuefei Zhou, Jingren Yang

**Affiliations:** 1 College of Environmental Science and Engineering, Tongji University, Shanghai, 200092 China; 2 Shanghai Academy of Environmental Sciences, Shanghai, 200233 China

**Keywords:** Dry anaerobic digestion, Biochar addition, Biogas production, Antibiotic resistance genes

## Abstract

To explore the bioaugmentation mechanism of biogas-production promotion and risk reduction of antibiotic-resistance genes (ARGs) in the anerobic co-digestion of cattle manure and rice straw with biochar addition, the performances of digestion and productivity with different amounts of biochar additions (0, 1.25, 3.75 and 5 g·L⁻¹ slurry) were studied. The results indicated that, biochar addition could effectively promote biogas production, and the cumulative methane (CH_4_) yields from the treatments with 3.75 g·L⁻¹ slurry and 5 g·L⁻¹ slurry biochar additions were18.7 times and 14.8 times of CK (0 g·L⁻¹ slurry), respectively. Combined with Fourier transform infrared spectroscopy (FTIR) analysis and high-throughput sequencing-based microbial community quantification, the methanogenesis was enhanced through three possible pathways: (1) the porous structure and aromatization characteristics of biochar could promote destruction of cellulose bundle structure, thereby promoting the hydrolysis of lignocellulosic substrates; (2) Biochar regulated volatile fatty acid (VFA) concentrations within an optimal range, enhancing the buffering capacity of the anaerobic digestion system; (3) The low-dose biochar (1.25 g·L⁻¹ slurry) achieving the optimal ARG risk mitigation by suppressing the primary ARG host *Bacteroidota*.

**Graphical Abstract:**

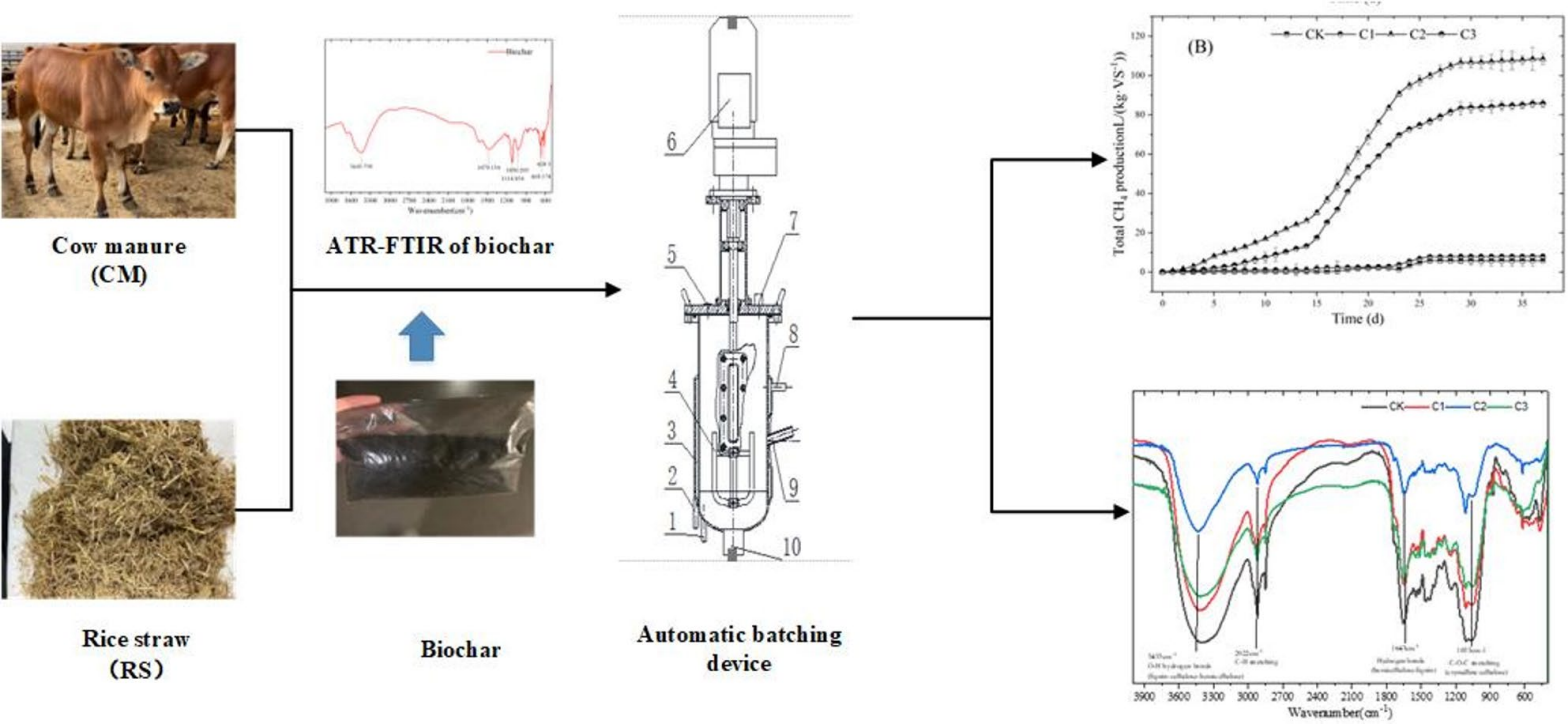

**Supplementary Information:**

The online version contains supplementary material available at 10.1186/s40643-025-01003-2.

## Introduction

Crop straw and animal manure are two of the most important types of organic waste. China produces over 800 million tons of crop residue each year, with rice straw primarily distributed in southern China (Li et al. 2015a; Zhang et al. 2020), and rice straw (RS) returning to the fields is a mainly disposal approaches in southern China nowadays, which is an effective measure to enhance resource utilization of rice straw, increase soil structure and nutrients (Zhuang et al. 2020). However, this approach would lead to serious environmental problems and increase greenhouse gas emissions, weakening the environmental benefits of this practice (Pramono et al. 2021). Furthermore, the total annual production of cow manure (CM) from large-scale centralized farms is about 382 million tons (Li et al. 2015a). The various policies in China introduced to encourage the return of livestock waste to the field, leading to many problems, such as mismatch of planting and breeding scale and difficulty in environmental supervision (Shi et al. 2018). As the ‘Peak Carbon and Net-zero Emission Goals’ (Double carbon goals) have become a national strategy, it is essential to limit greenhouse gas emissions from the land application of agricultural waste and transition to alternative low-carbon energy generation options(Khoshnevisan et al. 2021). Anaerobic co-digestion (ACD) of cow manure and rice strawis of vital importance to agricultural waste management and resource recycling around large-scale centralized farms (Surendra et al. 2014). Compared to wet anaerobic digestion (< 15% solids), dry anaerobic digestion exhibits significant economic benefits, such as less energy demands, less lignocellulosic biomass feedstock requirement and smaller reactor (Ge et al. 2016; Sun et al. 2019). Therefore, dry anaerobic co-digestion has emerged as a feasible and cost-effective approach for low-carbon energy generation from rice straw and animal manure.

Biochar is a carbon-rich material obtained via biomass pyrolysis at 500–700 °C under low oxygen conditions, and the properties of high specific surface area, porosity, and cation exchange capacity of biochar make it an efficient adsorbent material (Fagbohungbe et al. 2017; Sanchez-Monedero et al. 2018). Xie et al. (2021) showed activated carbon addition could promote the cellulose degradation rate and increase methane yield during the anaerobic digestion of cornstalk. Yang et al. (2021b) found that biochar added during anaerobic digestion enhance methanogenesis and affect the abundance of antibiotic resistance genes (ARGs) in swine manure. Crop straw is the common feedstock for straw carbonization (biochar production), which has been used to enhance the amount of phosphorus and activate the stable phosphorus for soil amendment to improve soil fertility (Fernandez-Bayo et al. 2020; Ma et al. 2019). However, the addition of biochar from rice straw carbonization during dry anaerobic digestion process has rarely been explored.

Meanwhile, the increasing prevalence and risk of ARGs in livestock and poultry manure through land application has attracted considerable attention (Yue et al. 2021). The residual antibiotics in livestock and poultry manure is the frequent spread of antibiotic resistance, including antibiotic-resistant bacteria and ARGs in the environment (Menz et al. 2019). The risks of antibiotic resistance are correlated with the mobile nature of ARGs, which could be transferred to organisms of different communities. ARGs may retain viability following host-cell death and recombine genetic material with living bacteria through transformation (He et al. 2016; Wallace et al. 2018). Apparently, livestock manure was a dominating source of some kinds of antibiotics and ARGs, which could enter the environment through land application (Tuo et al. 2018). Anaerobic digestion is generally regarded as an effective method to reduce ARGs in the digestates (Yang et al. 2020), and the biochar addition in the process could reduce ARGs dissemination simultaneously (Yang et al. 2021b). Thus, the digestate of anaerobic digestion process is a valuable form of organic fertilizer, and the risk assessment of ARGs during anaerobic digestion with biochar addition is necessary.

In this study, the effect of biochar addition on the productivity of Dry-ACD of CM and RS were studied with an automatic batching reactor, and the biogas production, the composition of the digestate (cellulose, hemicellulose, and lignin), Fourier Transform Infrared Spectroscopy (FTIR) analysis, and the quantitative assessment of microbial communities. The bioaugmentation mechanism in promoted biogas-production and reduced ARGs’ risk with biochar addition in Dry-ACD process were explored. According to the results, the possible optimal ratio of biochar addition was discussed, considering biogas production efficiency, digestate performance, and potential host bacteria abundance of ARGs.

## Materials and methods

### Substrates and biochar

The rice straw was collected from an agricultural park in Qingpu District, Shanghai, China. It was cut into 3–5 cm pieces and air-dried at ambient temperature. The biochar used in this study was prepared through hydrothermal carbonization (200 °C, 0.5 h) followed by pyrolysis (650 °C). The preparation procedure was as follows: first, rice straw was placed in a hydrothermal reactor under oxygen-limited conditions, heated to 200 °C, and maintained for 0.5 h. After cooling, the resulting hydrochar was transferred to a tubular furnace and heated to 650 °C at a rate of 10 °C·min^−1^, held for 0.5 h, and then cooled to room temperature. The obtained biochar was oven-dried at 105 °C for 12 h, ground with a disk-rotating mill, and sieved through a 100-mesh screen. The cow manure was acquired from a cattle farm in Jinshan District, Shanghai, China. The inoculum was prepared after solid-liquid separation from a dry anaerobic digestion plant in Qingpu District, Shanghai, China. The main physicochemical property of raw materials used in this study was listed in Table 1.

**Table 1:** Physical and chemical properties of Raw materials

Parameter	Rice straw	Cow manure	Inoculum	Biochar
TS (%)	86.8 ± 0.78	25.0 ± 0.50	4 ± 0.64	–
C (%)	42.12 ± 0.23	26.40 ± 1.00	45.32 ± 0.7	57.45 ± 2.00
N (%)	0.62 ± 0.03	1.20 ± 0.50	6.23 ± 0.20	0.61 ± 0.03
H (%)	5.92 ± 0.21	–	–	1.95 ± 0.5
Hemicelluloses (%)	19.23 ± 1.01	12.35 ± 0.09	–	–
cellulose (%)	41.53 ± 1.32	35.45 ± 1.34	–	–
lignin (%)	14.4 ± 0.96	13.5 ± 0.05	–	–
C/N ratio	68:1	22:1	7:1	–

All elemental contents (C, H, N) are expressed as % of dry matter (DM); moisture content of each matrix was determined by oven-drying at 105 °C to constant weight

### Dry-ACD batch experiment

**Fig. 1:**
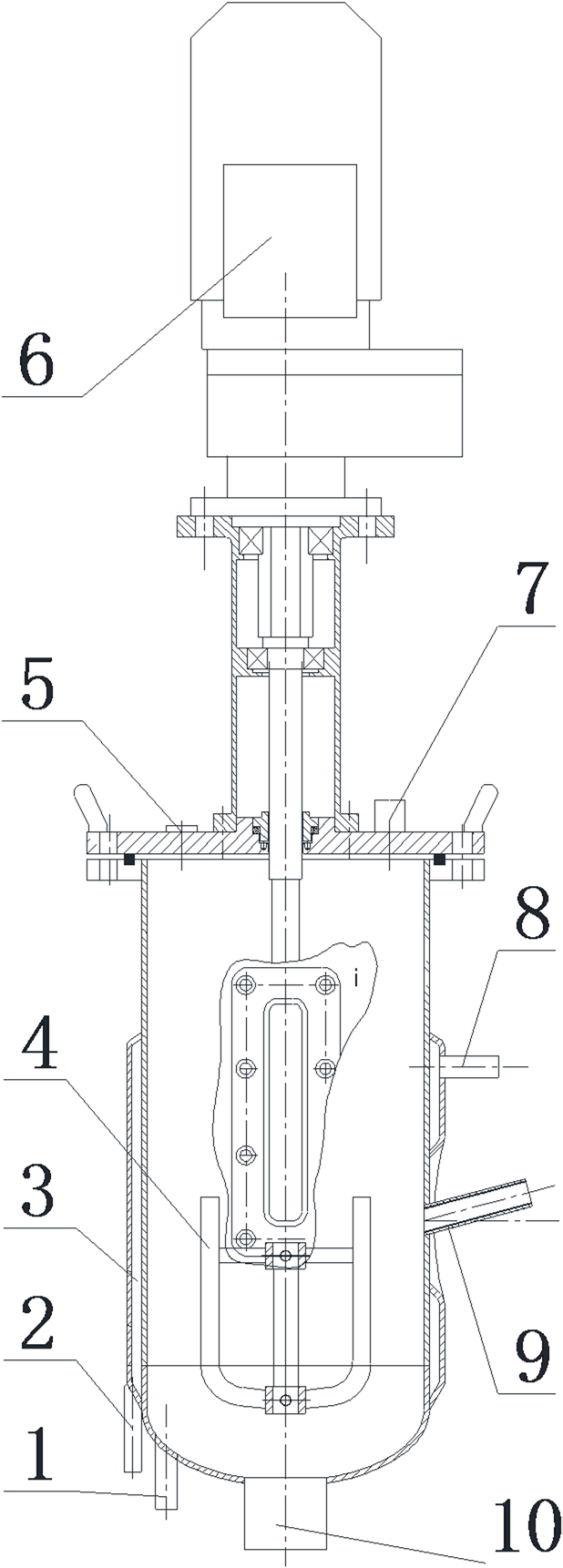
Sectional view of automatic batching device 1. Temperature monitoring probe 2. Inlet pipe 3. Temperature control water jacket 4. Stirring blade 5. Feed inlet 6. Electric motor 7. Exhaust port (connected to flow meter) 8. Outlet pipe 9. pH monitoring probe 10. Discharging port

The automatic batching reactor for Dry-ACD of CM and RS with biochar addition was presented in Fig. 1. The volume of a single digester tank is 5 L and there are four digester tanks in the device. The reactor is equipped with mechanical stirring system, water bath cycle insulation system, pH and temperature online monitoring system. In addition, the reactor is equipped with automatic PLC (computer control system), with real-time monitoring, operation controlling and data reading. A variable-speed paddle stirrer (rotational speed set at 60 rpm) is installed in each tank to ensure homogeneous mixing of the solid-liquid mixture. Stirring is programmed to operate in 15-min intervals (15 min on/15 min off) to prevent sedimentation of solid substrates (RS, CM) and biochar, while minimizing excessive aeration that could inhibit anaerobic microbial activity.

As specified in Table 2, four treatments (CK, C1, C2, C3) were designed with varying biochar dosages, while other key parameters were kept consistent. For each digester tank, RS (260 g TS) and CM (400 g TS) were mixed to achieve a target C/N ratio of 30 (actual ratios: 30.42–31.30, Table 2), leveraging the complementary C/N ratios of RS (high C) and CM (moderate N) to optimize microbial nutrient supply.

**Table 2:** Experimental design of dry anaerobic co-digestion of rice straw with cow manure and Biochar as addition

Treatments	RS (g (TS))	CM (g (TS))	Biochar (g·L⁻¹ slurry)	TS (%)	C/*N*	Inoculum (%)
CK	260	400	0.00	15	30.42	20
C1	260	400	1.25	15	30.64	20
C2	260	400	3.75	15	31.08	20
C3	260	400	5.00	15	31.30	20

RS, rice straw; TS, total solid; CM, cow manure; C/N, ratio of carbon and nitrogen; the basis of “%” was dry mass

As shown in Table 2, biochar was added to four digester tanks of the reactor at different dosages (0, 1.25, 3.75, and 5 g·L⁻¹ slurry), where the dosage refers to the mass of dry biochar per liter of digestion slurry. Biochar was added at dosages of 0 (CK, control), 1.25 (C1), 3.75 (C2), and 5.00 (C3) g·L⁻¹ slurry (dry biochar mass per liter of digestion slurry). The total volume of digestion slurry in each tank was adjusted to 4 L (to match the 5 L tank volume and 15% TS content), so the actual biochar masses added were 0 g, 5 g, 15 g, and 20 g per tank, respectively. The inoculum was added at 20% (dry mass basis), corresponding to 80 g TS of inoculum per tank, ensuring sufficient microbial biomass to initiate digestion. The fermenter tanks were then sealed and incubated, controlled by automatic thermostat water bath at 35 ± 0.5 °C for 38 days. Triplicate samples were collected from each tank on days 0, 5, 10, 15, 20, 25, 30, and 37 for offline analysis (e.g., TS/VS content, VFA concentration, microbial community sequencing), while biogas production was also monitored daily by an online monitoring sensor. To eliminate experimental errors, all treatments were conducted in triplicate, and blank controls (inoculum only, no substrates or biochar) were included to correct for biogas production from the inoculum itself.

### Analytical methods

#### Physicochemical properties

The daily biogas production for each digester tank was tested in real time by online gas flow meter and the methane content in the biogas was monitored by a portable CH_4_ detector every day (Shenzhen Keernuo, China). The contents of cellulose, hemicellulose, and lignin were analyzed based on the Van Soest method (Yan et al. 2015). The pH value and temperature in the digester tanks were recorded in real time by online pH glass gel electrode and online temperature sensor. The CH_4_ content was measured using a portable methane analyzer (Model: GASTiger2000, Shenzhen Wandi Technology Co., Ltd.). The total solid (TS) and volatile solid (VS) of the materials were determined by the standard detection methods (APHA 1998). The contents of total carbon and total nitrogen of raw material were analyzed with the elemental analyzer (Li et al. 2015b). Hemicellulose, cellulose, and lignin determination: Analyzed using the van Soest method (neutral detergent fiber (NDF), acid detergent fiber (ADF), and acid detergent lignin (ADL) sequential extraction(Syauqi et al. 2024). All the experiments were carried out in triplicate. FT-IR was used to analyze chemical properties of the digestates in different groups, and the spectra range from 400 to 4000 cm^− 1^ of the digestates.

#### Analysis of microbial community diversity

Samples was extracted from biogas residues and cow manure selected for microbial community analysis. The bacteria 16S ribosomal RNA gene in V1-V9 regions were amplified by PCR and the DNA Kit (Omega Bio-tek, Norcross, GA, U.S.) was used. The PCR used primers 27F 5’-AGRGTTYGATYMTGGCTCAG-3’and1492R 5’-RGYTACCTTGTTACGACTT-3’ and the detail of that treatment was as follow: 95 °C for 2 min, later on 27 cycles at 95 °C for 30 s, 55 °C for 30 s, and 72 °C for 60 s and the last extension at 72 °C for 5 min).PCR reactions were performed in triplicate 20 µL mixture containing 4 µL of 5 ×FastPfu Buffer, 2 µL of 2.5 mM dNTPs, 0.8 µL of each primer (5 µM), 0.4 µL of FastPfu Polymerase, and 10 ng of template DNA. Amplicons were extracted from 2% agarose gels and purified using the AxyPrep DNA Gel Extraction Kit (Axygen Biosciences, Union City, CA, U.S.) according to the manufacturer’s instructions.

SMRTbell libraries were prepared from the amplified DNA by blunt-ligation according to the instructions from the manufacturer (Pacific Biosciences). Purified SMRTbell libraries from the pooled and barcoded samples were sequenced on a single PacBio Sequel II cell. All amplicon sequencing was performed by Shanghai Biozeron Biotechnology Co. Ltd (Shanghai, China).

The rarefaction analysis based on Mothur v.1.21.1 was conducted to reveal the diversity indices, including the Chao, ACE, and Shannon diversity indices (Schloss et al. 2009). One-way analysis of variance (ANOVA) followed by Tukey’s post-hoc test was used to compare alpha diversity indices among groups (*p* < 0.05).

Core phyla with a relative abundance ≥ 1% in at least one treatment group were screened out. Subsequently, the association between these phyla and the functional genes carried by ARG hosts was validated based on published literature and the CARD database (Shan et al. 2023; Yang et al. 2021a; Zhang et al. 2023). Low-abundance phyla (< 0.5%) or those without functional evidence of being ARG hosts were excluded from ARG risk analysis. Based on the functionally validated ARG detection probability, the ARG detection risk index was calculated as follows:

$$ \begin{aligned}{\text{ARG }}\;{\mathrm{Detection}}\;{\text{ Risk }}\;{\mathrm{Index}} & = \sum ({\text{Phylum }}\;{\mathrm{Relative}}\;{\text{ Abundance}} \\ & \quad \times {\mathrm{Functionally}}\;{\text{ Validated}}\;{\text{ ARG}}\;{\text{ Detection}}\;{\text{ Probability}}) \end{aligned}$$

Pearson correlation analysis was performed to assess the relationship between core phylum abundance and ARG potential risk (*n* = 4, significance level *p* < 0.05).

## Results and discussion

### Biogas production

**Fig. 2:**
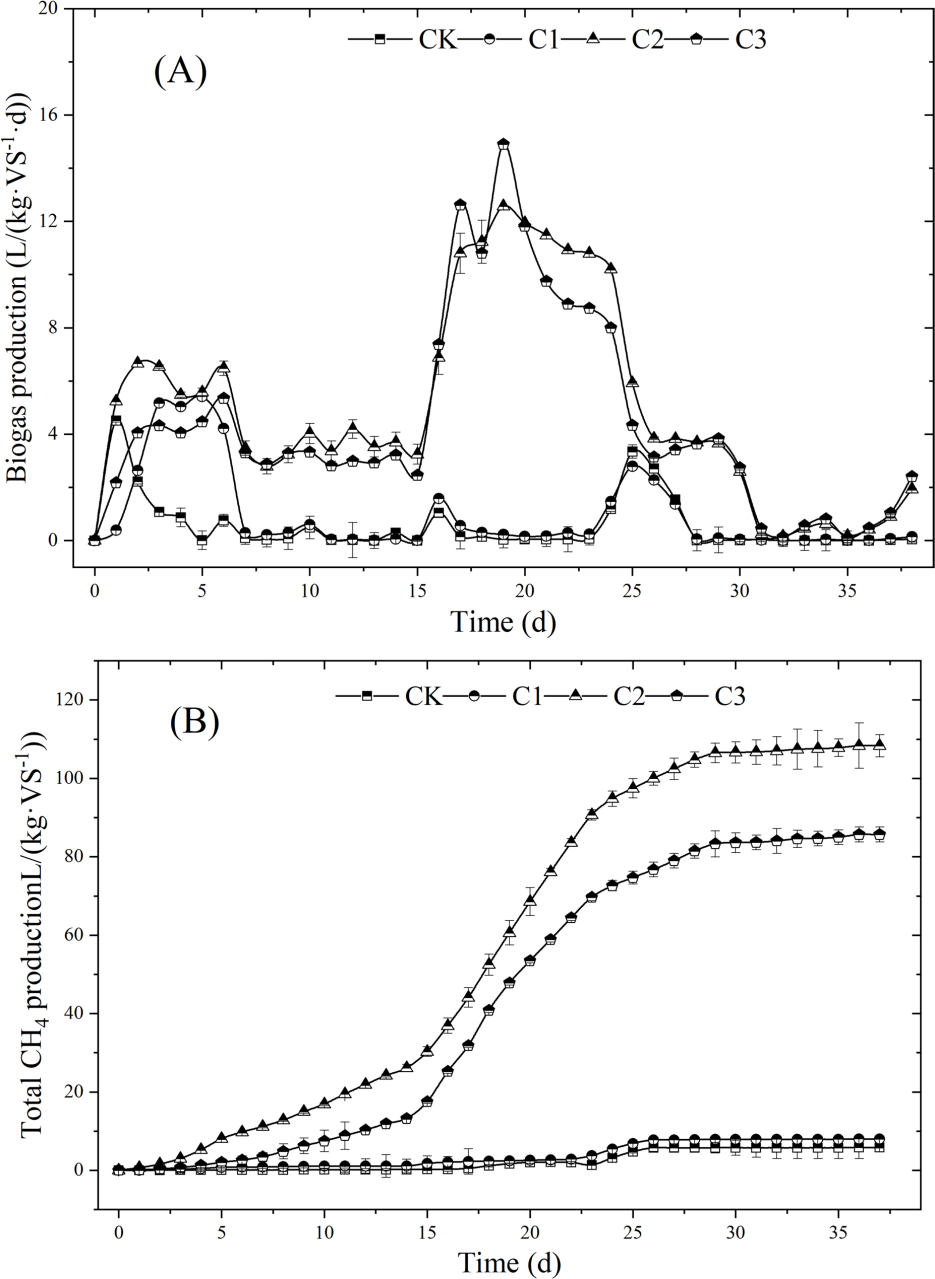
The daily biogas production (**A**) and the cumulative CH_4_ yields (**B**) of different treatments

The daily biogas production of different treatments with biochar additions during Dry-ACD process is shown in Fig. 2A. The biogas production of all the treatments was mainly concentrated in the second peak in the whole digestion process, while the first peaks of these treatments only about 2–6 days, and digestion process would be at the hydrolysis stage during the first peak. The second peak of these treatments was methanogenesis stage, with the methane contents at this stage were in the range of 50–65% (as shown in Fig. S1), and the digestion could be in the state of balance, and supply biogas with stable methane content (Zhang et al. 2015).

The biogas production was significantly different with the different proportion of biochar addition. The first peak of lignocellulosic biomass hydrolysis in CK appeared on day 2, and then biogas production declined rapidly and entered into lag period on day 5, while the first peaks of hydrolysis in C1, C2 and C3 were all delayed to day 8. Compared to CK, the durations of the first peaks in C1, C2 and C3 were obviously prolonged, which indicated the hydrolysis were enhanced by facilitating the degradation of lignocellulosic substances with biochar addition. Biogas production achieved peak values at day 16 in C2 and C3, which would help in ensuring stable biogas supply about 15 days, while the peak values in CK and C1 was delayed to day 19–20, with the shortened duration of peak values of 10 days. This might be explained that, biochar addition conduced to methane production, and microorganism growth helped shorten the time to generate biogas and increase the stable biogas supply time (Yang et al. 2021b). Thus, a suitable dosage of 3.75–5 g·L⁻¹ slurry biochar addition (based on TS) could increase biogas yield and reduce hydrolytic acidification lag phase time.

The cumulative CH_4_ yields were shown in Fig. 2B, and the cumulative biogas yields in C2 and C3 were much higher than other treatments. In the whole digestion process, the cumulative biogas yields in CK and C1 were only5.78 L/(kg·VS^− 1^) and 7.97 L/(kg·VS^− 1^), while that of C2 and C3 were 18.7 times and 14.8 times of CK. This result indicated that, the biogas production had been obviously inhibited in CK and C1, which probably because the mass transfer efficiency was suppressed, and caused low pH and interfere with substrate biodegradation, then influences transformation efficiency and limits the biogas production (Fagbohungbe et al. 2017). Therefore, the biochar addition can improve the reaction stability by adsorbing inhibitors such as volatile fatty acids (VFAs), ammonia, and limonene, which subsequently shortened the lag period and improved gas production (Pan et al. 2019). Consequently, appropriate amount of biochar addition could promote methane production and microorganism growth, and alleviate inhibition to increase biogas production. In this study, the C2 with 3.75 g·L⁻¹ slurry biochar addition was the optimal treatment to generate biogas efficiently.

### Change of VFAs and pH

**Fig. 3:**
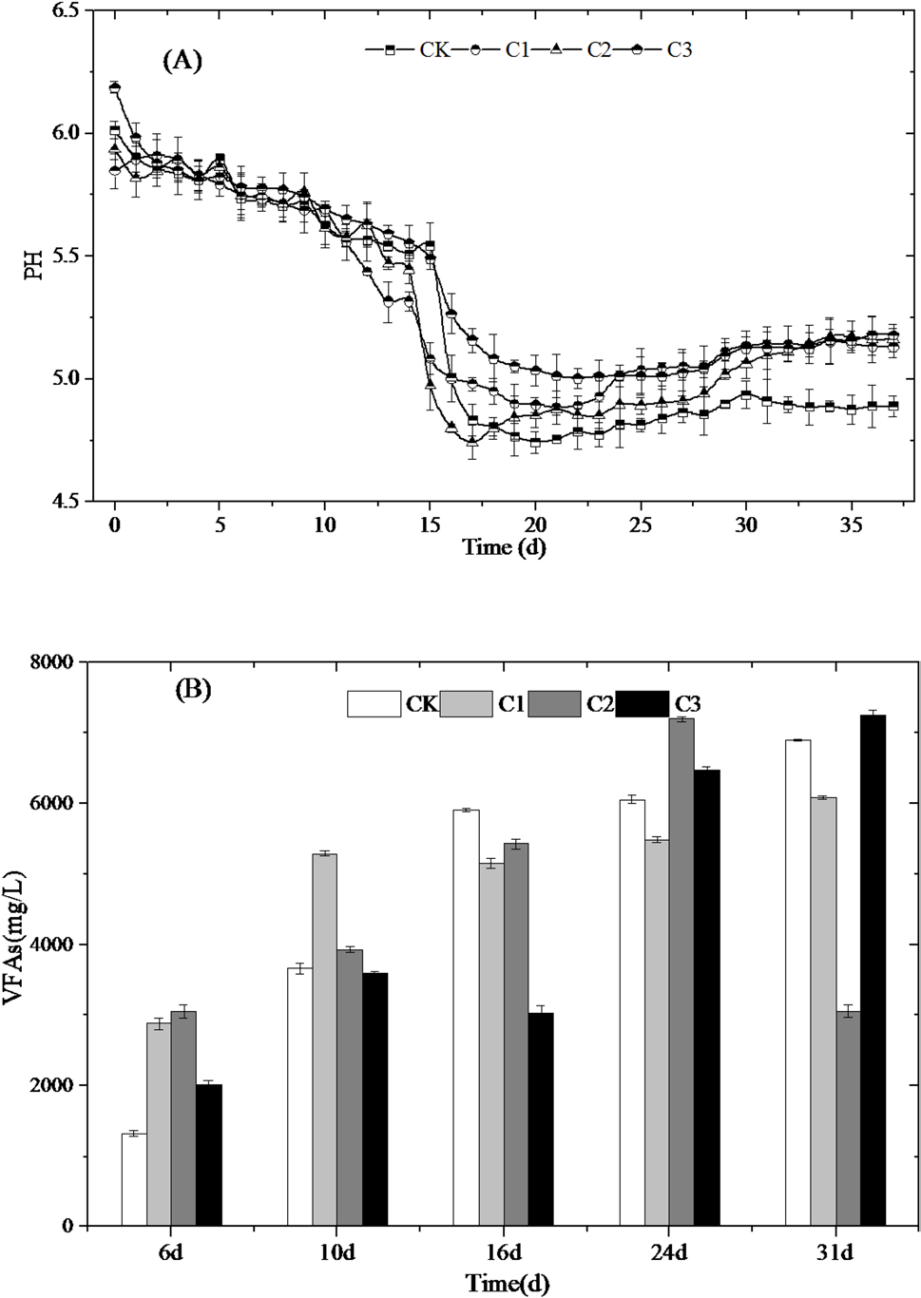
The changes of pH and VFAs of different treatments; **A** PH; **B** VFAs

Figure 3 showed the tendencies of pH and VFAs concentration changes in all treatments. The pH values were between 5.0 and 5.7, and the pH value of C2 quickly fell to 4.7 on day 16, then quickly reached to 5.3 on day 22, while that of CK slowly fell to 4.7 on day 18, then remained around 5.1. The low pH (< 5.5) phenomenon in this study is an inherent characteristic of the system—due to the higher dosage of cow manure than rice straw, the substrate is rich in readily degradable organic matter (such as soluble carbohydrates and proteins), leading to a high acidogenesis rate in the early stage (Hu et al. 2025). Additionally, no external buffers were added to avoid interfering with the efficacy of biochar, and the rapid accumulation of VFAs exceeded the initial buffering capacity of the system, ultimately resulting in the formation of an acidic environment. The results revealed obvious acid inhibition with in CK (no biochar addition), and the inhibition were alleviated in C2 by biochar addition, consist with the performance of biogas production. The opposite tendencies of pH and VFAs concentration changes indicated that, the decreases of pH values were just caused by the VFAs production (Li et al. 2015a). The concentrations of VFAs in different treatments were 2020–3060 mg/L, higher than that in CK, which was 1323 mg/L on day 6. After 16 days, the concentrations of VFAs in the treatments with biochar additions decreased remarkably. Previous studies found that the high concentration of VFAs is not benefit for subsequent methane production, and the cellulose degradation could be inhibited at the concentration above 2000 mg/L (Zhang et al. 2016). These results indicated that, VFAs were produced from cellulose degradation before day 16, and biochar addition might have contributed to cellulose degradation. After day 16 at methanogenic stage, the VFA concentration in C2 was above 2000 mg/L, benefit for methane production and the cellulose degradation, while the VFA concentration in C3 was above 6000 mg/L, inhibiting methane production. Thus, a suitable dosage of 3.75–5 g·L⁻¹ slurry biochar could maintaining volatile fatty acids (VFAs) at a moderate concentration to avoid acidification, while relying on the system’s inherent alkalinity (e.g., bicarbonate, carbonate) to sustain stable buffering capacity.

### Digestate performance analysis

#### Degradation of cellulose, hemicelluloses and lignin

**Fig. 4:**
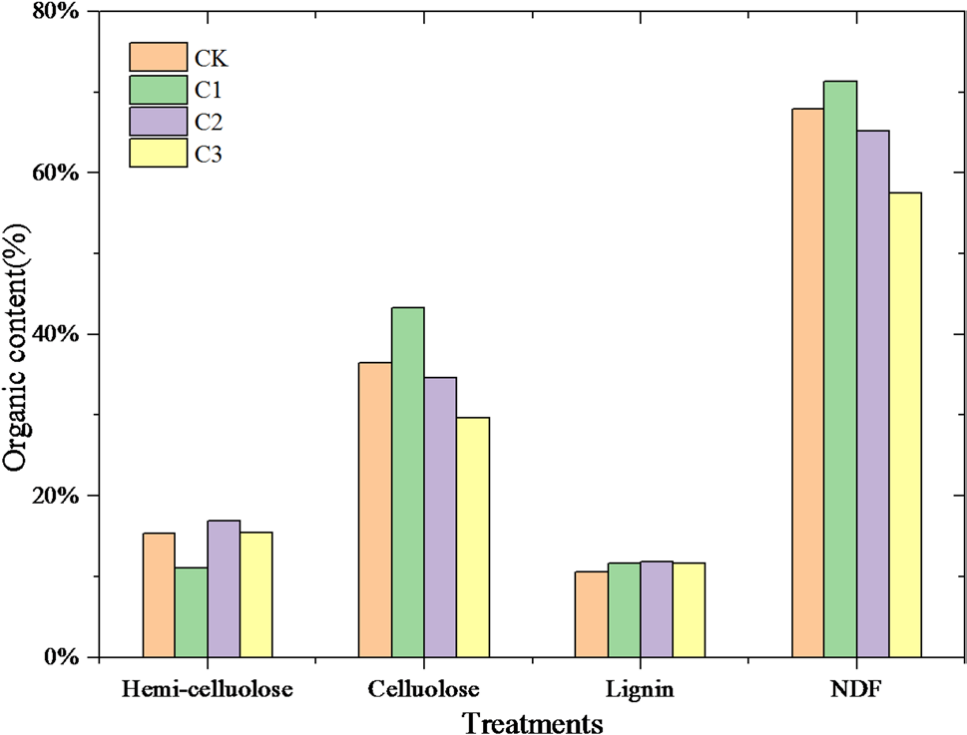
The contents of cellulose, hemicelluloses and lignin of digestates after anaerobic digestion

The contents of cellulose, hemicelluloses, lignin and neutral detergent fibers (NDF) in the digestates during the Dry-ACD process were presented in Fig. 4. The degradation efficiencies of rice straw and cow manure were determined, and the effect of biochar addition on degradation was evaluated. The content of NDF in C1 was 71.38%, which were 1.09 and 1.27 times of C2 and C3, indicated that the lignocellulosic degradation rates were significantly increased by biochar addition. Therein, the contents of lignin in different treatments were in the range of 10.57–11.88%, since lignin is harder to degrade than lignocellulosic and cellulosic in anaerobic conditions (Du et al. 2019). However, the contents of hemicelluloses and cellulose had significant difference. The hemicellulose content of Group C1 (11.67%) was significantly lower than that of the other groups (15.31% for CK, 16.86% for C2, and 15.45% for C3), suggesting that hemicellulose may be a relatively more easily degradable component under the condition of insufficient biochar addition (Group C1). The content of cellulose in CK was 36.45%, while that of C2 and C3 was 34.58% and 29.61%, indicating the significant degradation of cellulose with increasing biochar contents. Therefore, the sources of biogas production in C2 and C3 were celluloses degradation, consist with the conclusion of optimal biogas production.

**Fig. 5:**
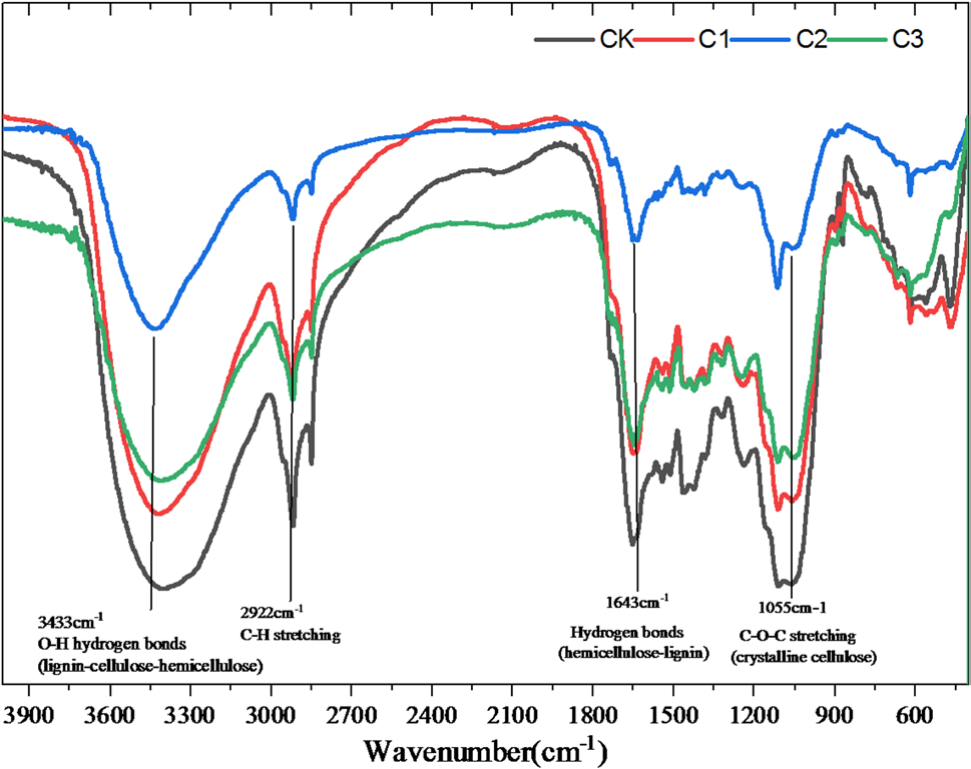
FT-IR spectroscopy of the functional groups among digestates in different groups

In order to verify the enhancement of celluloses degradation by biochar, the FT-IR analysis was adopted to test the functional groups among digestates in different treatments (Fig. 5). The characteristic peaks of C2 and C3 markedly weakened compare to C1 and CK at 3433 cm^− 1^, of which the absorption peak was hydrogen bond between lignin, cellulose and hemicellulose (Ao et al. 2020; Luo et al. 2018). This appearance presented that biochar addition could efficiently enhance the destruction of cellulose bundle structure. The characteristic peak at 1648 cm^− 1^ in C1 was weaker then that absorption peaks between C2 and C3. The characteristic peak at 1648 cm^− 1^ was the hydrogen bond between hemicellulose and lignin (Jiang et al. 2015), which was consistent with the result that the source of biogas production in C1 was hemicelluloses degradation. Besides, the peak at 1055 cm^− 1^ was significantly weakened in C2, indicating the degradation and destruction of crystal structure (Jiang et al. 2015). Compared to CK, the peaks of different treatments with biochar addition were changed further, and the peak was the weakest especially for C2. It could be concluded that, biochar addition can promote crystalline cellulose degradation, accordant with the result of cellulose degradation.

To fully verify the enhancement effect of biochar on cellulose degradation, a semi-quantitative cellulose/lignin ratio (using the intensity ratio of the cellulose characteristic peak at 1050 cm⁻¹ to the lignin characteristic peak at 1643 cm⁻¹ as the core index) was introduced based on functional group peak analysis. Combined with peak position changes, this enabled multi-dimensional interpretation of the lignocellulose degradation mechanism. The cellulose/lignin ratios of CK, C1, C2, and C3 are 0.47, 0.56, 0.86, and 0.73, respectively. The C2 group exhibited the highest ratio (0.8653), an 84.7% increase compared to CK. This fully aligns with previous findings (weakening of the 1055 cm⁻¹ crystalline cellulose peak and destruction of the 3433 cm⁻¹ hydrogen bond peak), further confirming that C2 is the optimal scheme for promoting selective cellulose degradation. An elevated cellulose/lignin ratio indicates reduced residual cellulose content per unit mass of lignin—meaning the cellulose degradation rate is significantly higher than that of lignin. Because lignin has a stable structure and strong resistance to degradation, while biochar accelerates cellulose degradation and consumption by disrupting its aggregated structures (e.g., hydrogen bond networks, crystalline regions), leading to a ratio increase.

### Effect of Biochar addition on microbial community into promoted biogas-production

#### Bacterial community in each group

**Fig. 6:**
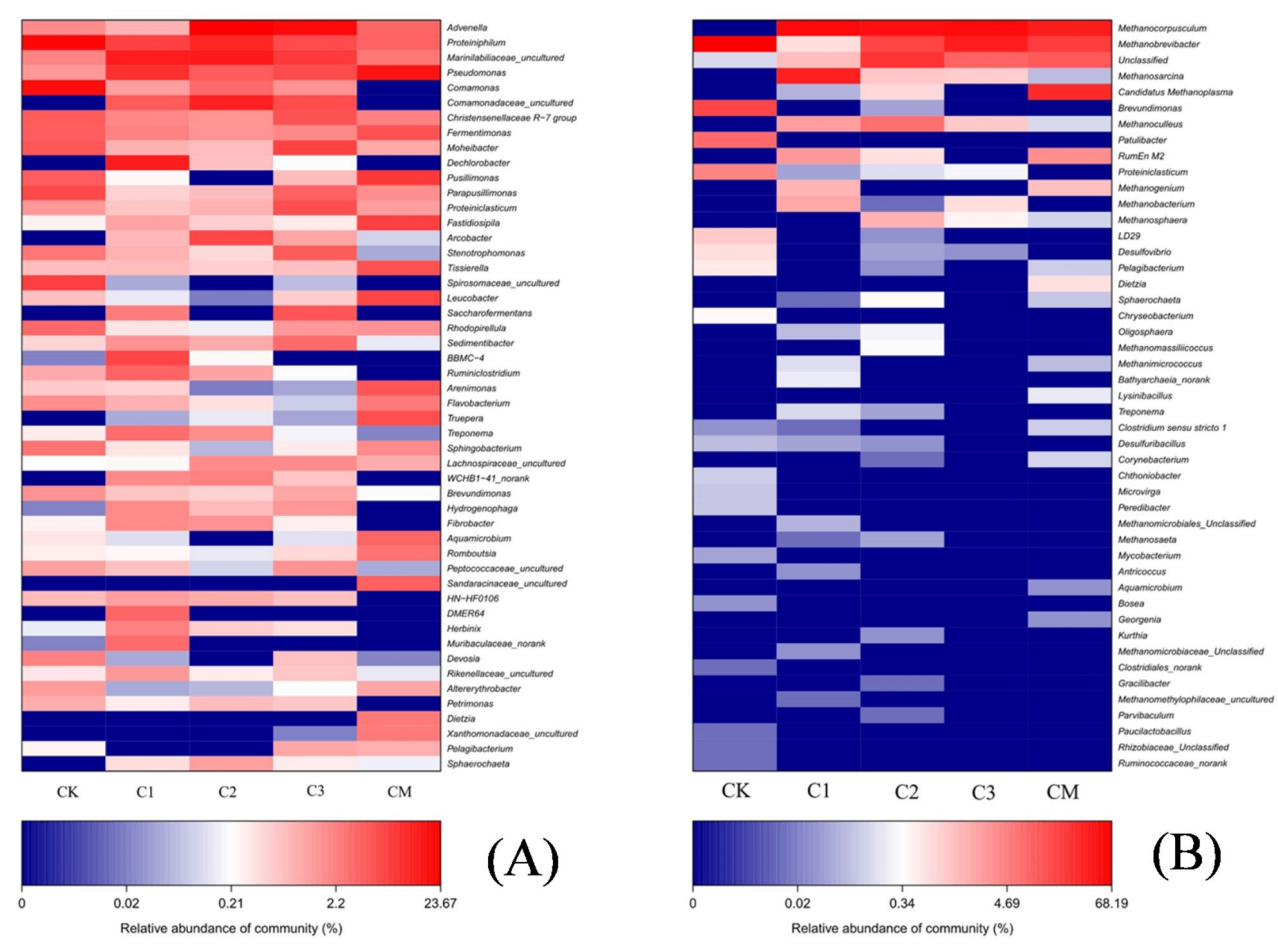
The community composition and difference of bacteria (**A**) and archaea (**B**) at the genus level in each group

Following the discussion of above physicochemical properties, the biochar addition could enhance cellulose degradation. To explore the causes of this phenomenon, the relative abundance of bacteria community (genus level) in all treatments was investigated, as showed in Fig. 6A. In CM, the major genera were *Pseudomonas* (11.93%), *Pusillimonas* (6.24%), *Fastidiosipila* (5.44%), *Truepera* (4.05%), *Fermentimonas* (3.90%), *Tissierella* (3.81%), *Arenimonas* (3.47%), *Aquamicrobium* (2.72%), *Advenella* (2.61%). By comparing the changes of microbial communities, the microbial communities’ diversity in all treatments (CK, C1, C2 and C3) increased and had all the dominant bacteria of CM. Therein, the *Advenella*, *Proteiniphilum* and *Marinilabiliaceae* increased remarkably, and their relative abundance in C2 could achieve to 20.39%, 12.57% and 9.69%, while the relative abundance of other dominant bacteria in C2 fell less to 1%. The abundance of *Ruminofilibacter* was the main utilize xylan (Krober et al. 2009), and HN-HF0106 could ferment cellulose and then degrade saccharides to H_2_ and acetate (Zhang et al. 2018). The relative abundances of *Ruminiclostridium* in CK, C1, C2 and C3 were 0.80%, 2.83%, 1.02% and 0.18%, while HNHF0106 were 0.62%, 0.89%, 0.84% and 0.58%, respectively, which had no exist in CM. It also indicated that the cellulose of C2 had a highest degradation rate due to microbial community variation. Combined with bacterial community results, the reinforcement effect on biogas production in the Dry-ACD process as a result of the enrichment of cellulolytic bacteria and concentrated carbon metabolize to methane.

#### Archaea community in each group

Promoted cellulose degradation suggested that more metabolic substrates could be used for methanogens, which would contribute to methane production (Xie et al. 2021). In order to find the key factor to promote the biogas yields, the archaea community in different treatments was monitored (Fig. 6B). The relative abundance of *Methanothermobacter* was 68.19% and there is no other methanogen in CK. But with biochar addition, the relative abundance of *Methanothermobacter* decreased to 0.68%, 16.40% and 36.12% in C1, C2 and C3, while the *Methanocorpusculum* increased to 55.03%, 47.48% and 51.54%, respectively. The relative abundance of *Methanothermobacter* and *Methanocorpusculum* were 18.70% and 37.25% in CM, indicating the biochar addition directly enriched *Methanocorpusculum* and weakened *Methanothermobacter*. *Methanothermobacter* and *Methanocorpusculum*, which were common in cattle manure, and functioned as hydrogenotrophic methanogen, consist of H_2_ and CO_2_ consumption to produce CH_4_ (Ao et al. 2019). According dominant genera changes of methanogen, the biochar added to anaerobic digestion system could promote methanogenic performance and promote methane production through the direct enrichment of *Methanocorpusculum*.

Furthermore, the relative abundance of *Methanoculleus*, *Methanosarcina* and *Methanosaeta* also had a further increase in the treatments with biochar addition. *Methanoculleus* is not only a hydrogenotrophic methanogen, but also has specific function in the cellulose-rich anaerobic digestion system (Li et al. 2020). The specific function of *Methanoculleus* and the change of relative abundance of *Methanoculleus* were the other two important reasons to improve methane production. *Methanosarcina* could utilize acetate, H_2_/CO_2_, and methyl organics to produce methane in anaerobic digestion system (Fernandez-Bayo et al. 2020). *Methanosaeta* are acetolactic methanogens that convert acetate to methane (Shintani et al. 2021). Thus, a better performance of methane production might be attributed that, the biochar addition could enhance the abundance of *Methanosarcina*, *Methanosaeta* and *Methanoculleus*, covering a variety of methanogens.

### ARGs risks of Biowaste during dry anaerobic co-digestion with Biochar addition

The richness and diversity of community were the driving factors impacting ARGs during dry anaerobic co-digestion process (Wallace et al. 2018; Zhang et al. 2019). Table 3 showed Alpha diversities of microbial communities during anaerobic co-digestion, the sequences obtained from different treatments covered 93–98% of the species, thereby demonstrating that the microbial community was analyzed comprehensively (Sun et al. 2019). The Chao1 and ACE indices are core indicators reflecting community richness. The results show that the richness indices of C1 (Chao: 2233.91, ACE: 2497.17), C2 (Chao: 2302.04, ACE: 2568.66), and C3 (Chao: 2089.00, ACE: 2263.48) groups are significantly higher than those of the CK group (Chao: 1349.58, ACE: 1414.18), with statistically significant differences (Chao: *P* = 0.000, ACE: *P* = 0.000). This suggests that biochar addition is beneficial for improving microbial community richness. The Shannon index is used to comprehensively evaluate community richness and evenness. The C1 group has the highest Shannon index (8.98), and with the increase in biochar addition, the indices of C2 (8.40) and C3 (7.93) groups gradually decrease, with significant differences between groups (*P* = 0.011*). The results showed that the richness and diversity of community increased due to a certain amount of biochar addition, but the excessive content of biochar would inhibit the richness and diversity of community.

**Table 3:** Alpha diversities of microbial communities during anaerobic co-digestion

Treatment	Chao	Richness	Shannon	ACE	coverage
CK	1349.58	1223	8.23	1414.18	0.98
C1	2233.91	1815	8.98	2497.17	0.93
C2	2302.04	1948	8.40	2568.66	0.93
C3	2089.00	1472	7.93	2263.48	0.95
*P*-value	0.000	0.009	0.011	0.00	0.81
Significance	***	**	*	***	ns

*** (*p* < 0.001);** (*p* < 0.01);* (*p* < 0.05); ns (*p* ≥ 0.05)

**Fig. 7:**
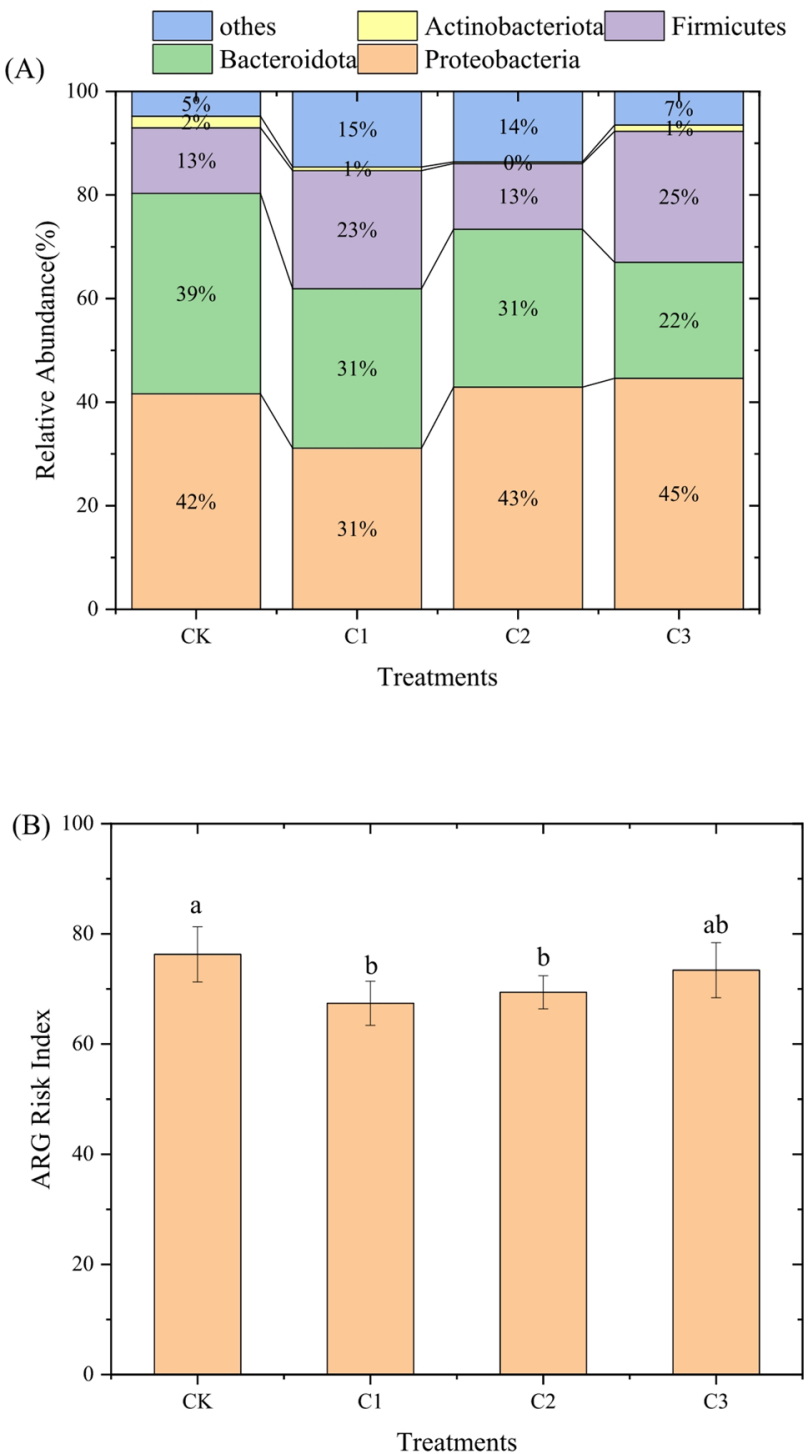
ARGs risks of biowaste during dry anaerobic co-digestion with biochar addition. Relative Abundance (**A**); ARGs Risk index (**B**)

As shown in Fig. 7A, the anaerobic co-digestion (AcoD) system was dominated by six core phyla, among which four major phyla were Proteobacteria (31.1–44.6%), Bacteroidota (22.4–38.7%), Firmicutes (12.7–25.3%), and Actinobacteriota (0.3–2.2%). The relative abundance of Bacteroidota decreased with increasing biochar dosage: it was 38.7% in the CK group, whereas it dropped to 22.4% in the C3 group. The relative abundance of *Actinobacteriota* in the C2 group was 0.29%, which was significantly lower than that in the CM group (2.2%). For Firmicutes, its abundance increased in the biochar-amended groups C1 and C2, reaching 22.8% and 25.3% respectively, compared with only 12.7% in the CK group. In terms of Proteobacteria, its abundance was the lowest in C1 (31.1%) and the highest in C3 (44.6%). Biochar amendment significantly reshaped the bacterial community structure in anaerobic co-digestion systems, which is consistent with previous findings(Li et al. 2022). The decrease in *Bacteroidota* abundance with increasing biochar dosage may be attributed to the porous structure of biochar, which can adsorb substrates preferred by *Bacteroidota* (e.g., complex carbohydrates)(Huang et al. 2024). In contrast, Firmicutes—a key acidogenic phylum—exhibited increased abundance in biochar groups, which may enhance volatile fatty acid (VFA) production and thereby promote methane generation (Liu et al. 2022). The resilience of Proteobacteria, which dominated in all treatments, suggests that it is less sensitive to biochar perturbation, possibly due to its metabolic versatility. The potential risk index of antibiotic resistance genes (ARGs) varied significantly among different treatments (Fig. 7B). Specifically, the control group (CK) exhibited the highest risk index (77.48), while the low biochar dosage group (C1) achieved the lowest risk index (68.79), representing an 11.2% reduction compared to CK. In contrast, the risk indices of the C2 (70.78) and C3 (75.73) groups were higher than that of C1, indicating a dosage-dependent threshold effect.

Pearson correlation analysis further revealed strong associations between the abundance of core phyla and ARG potential risk (Fig. S2). *Bacteroidota* showed a significantly strong positive correlation with ARG risk (*r* = 0.92, *p* = 0.028), suggesting it acts as the primary driver of ARG risk. Proteobacteria also exhibited a strong positive correlation with ARG risk (*r* = 0.79, *p* = 0.091), which approached statistical significance (*p* < 0.1). However, Firmicutes (*r* = 0.08, *p* = 0.905) and *Actinobacteriota* (*r* = 0.67, *p* = 0.168) showed no significant correlations with ARG risk. Notably, the low-dose biochar treatment (C1) was the most effective in mitigating ARG potential risks. Biochar reduced the abundance of *Bacteroidota*, which was identified as the primary ARG host (*r* = 0.92, *p* = 0.028), thereby decreasing the total ARG pool in the system. The increased ARG risk in the high-dose biochar groups (C2/C3) might be attributed to the enrichment of Proteobacteria (a secondary ARG host), which offsets the mitigation effect of reduced *Bacteroidota* abundance. This finding emphasizes the importance of optimizing biochar dosage to balance microbial community regulation and ARG risk mitigation in related systems.

## Conclusion

In the Dry-ACD of CM and RS, a suitable dosage of 3.75–5 g·L⁻¹ slurry biochar addition (based on TS) was found to be the key factor to promote biogas yield. The methanogenesis was enhanced, since the porous structure and aromatization characteristics of biochar could promote the destruction of cellulose bundle structure and that regulated volatile fatty acid (VFA) concentrations within an optimal range, enhancing the buffering capacity of the anaerobic digestion system. Biochar amendment increases microbial community richness and diversity at appropriate dosages while reshaping bacterial community structure (reducing *Bacteroidota* abundance and increasing Firmicutes abundance), with low-dose biochar (C1) achieving the optimal ARG risk mitigation (11.2% reduction vs. CK) by suppressing the primary ARG host *Bacteroidota* (*r* = 0.92, *p* = 0.028), though high doses (C2/C3) reduce mitigation efficiency due to Proteobacteria enrichment.

## Supplementary Information

Below is the link to the electronic supplementary material.

Supplementary Material 1

## Data Availability

The data and materials were presented in the manuscript and supplemental materials.
