# Supplementary material for: Mechanistic insights into promoted biogas production and reduced antibiotic resistance genes’ risks of dry anaerobic digestion of organic wastes with biochar addition
Source: Bioresour Bioprocess. 2026 Jan 27;13(1):7. doi: 10.1186/s40643-025-01003-2 (PMC12834851; doi:10.1186/s40643-025-01003-2)
Supplement: Supplementary file 1 — Supplementary Material 1 [file 40643_2025_1003_MOESM1_ESM.docx]

**Mechanistic insights into promoted biogas-production and reduced** **antibiotic resistance genes risks of biowaste** **dry anaerobic co-digestion with biochar addition**

Zhenqi Wang ^1,2^, Min Zhang ^2^*, Xiaoyong Qian^2^*, Yuanzhi Ni^2^, Xuefei Zhou^1^, Jingren Yang^2^

1College of Environmental Science and Engineering, Tongji University, Shanghai 200092, China

2Shanghai Academy of Environmental Sciences, Shanghai 200233, China

*Corresponding author. Email: qianxy@saes.sh.cn


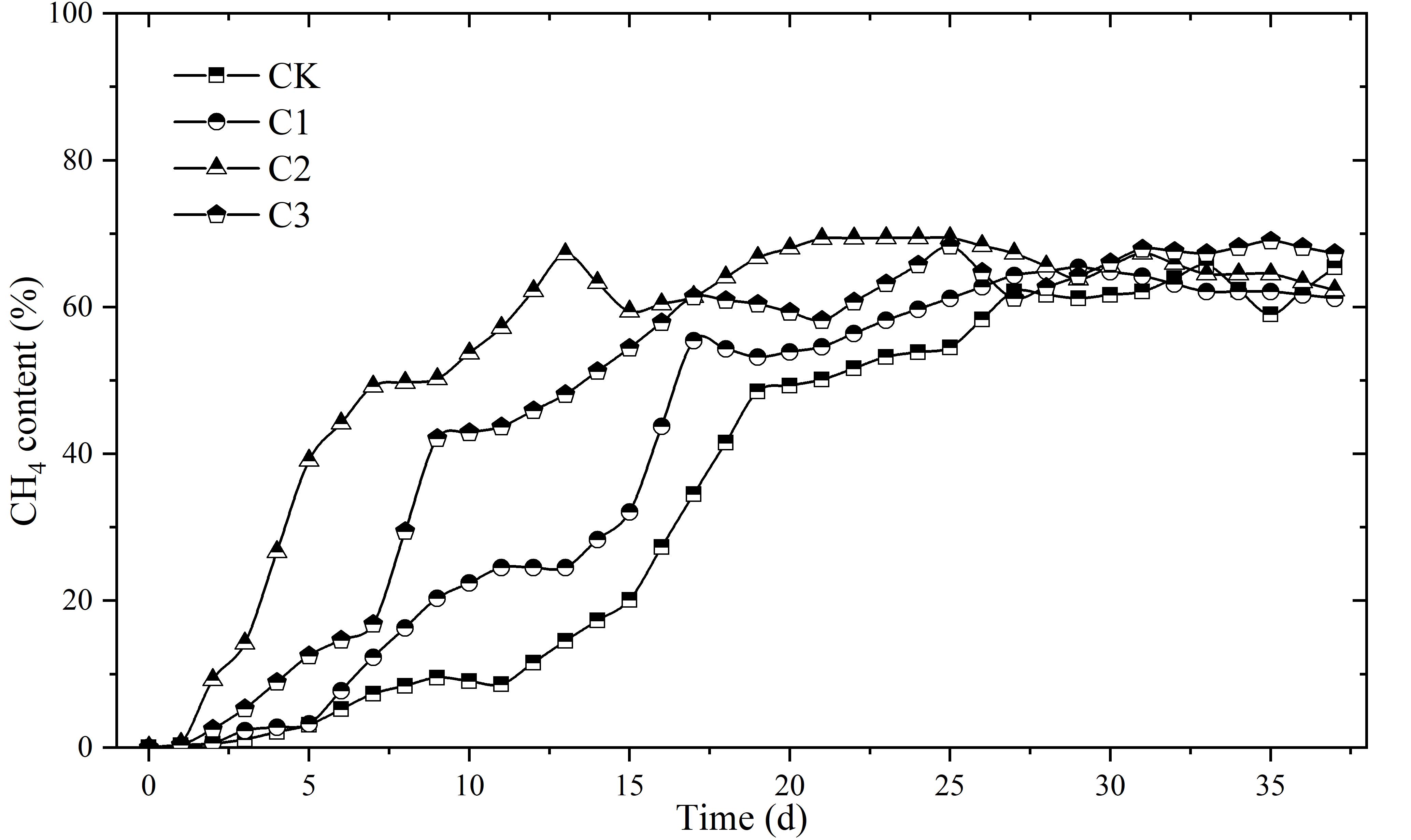


Fig. S1. Daily Methane Percentage Content Across Different Treatment Groups


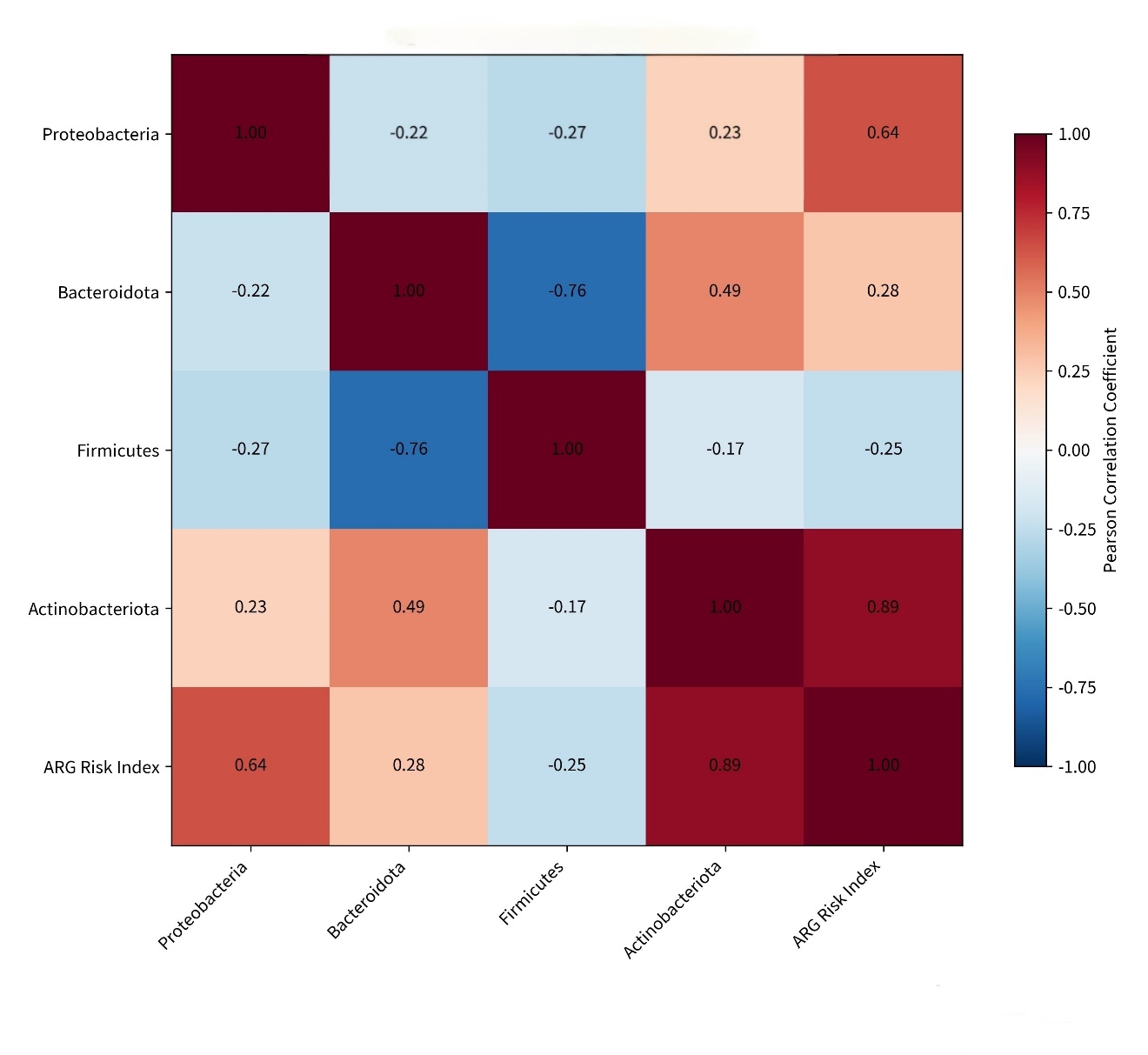


Fig. S2. Pearson correlation analysis was performed to assess the relationship between core phylum abundance and ARG potential risk.
